# An Interactive Haptic Guidance System for Intuitive Programming CNC Machine Tool

**DOI:** 10.3390/s21113860

**Published:** 2021-06-03

**Authors:** Kamil Stateczny, Karol Miądlicki

**Affiliations:** Department of Mechanical Engineering and Mechatronics, West Pomeranian University of Technology, Szczecin, al. Piastów 19, 70-310 Szczecin, Poland; kamil.stateczny@zut.edu.pl

**Keywords:** CNC control, machining assistance, operator, HMI, force control, haptic devices, programming by demonstration, force sensor, force measurements

## Abstract

The human-machine interfaces in modern CNC machine tools are not very intuitive and still based on archaic input systems, i.e., switches, handwheels, and buttons. This type of solution has two major drawbacks. The pushed button activates the movement only in one direction and is insensitive to the amount of the force exerted by the operator, which makes it difficult to move the machine axes at variable speeds. The paper proposes a novel and intuitive system of manual programming of a CNC machine tool based on a control lever with strain-gauge sensors. The presented idea of manual programming is aimed at eliminating the need to create a machining program and at making it possible to move the machine intuitively, eliminating mistakes in selecting directions and speeds. The article describes the concept of the system and the principle of operation of the control levers with force sensors. The final part of the work presents the experimental validation of the proposed system and a functionality comparison with the traditional CNC control.

## 1. Introduction

CNC designers are at a crossroad, with increasing demands for faster and flawless machining processes without increasing the skills requirements for CNC operators. Introducing too advanced and complex structures with a high level of abstraction in the CNC programming language, such as in modern, object-oriented programming languages, can cause many difficulties for the average CNC machine tool operator. There is therefore a tendency for the development of programming languages for CNC machine tools to simplify programming languages or the whole programming process for the CNC machine tool. Different types of workpiece visualizations or graphical simulations of the machining program are introduced to create easier more flexible environments. Control systems are connected to a computer network to transfer machining programs and exchange data with databases. These databases can store geometric parameters of tools located in the tool room and other data used in the technological machining process.

The complexity of solutions used in CNC machine tools is still growing new methods of surface quality improvement [1] and topographic inspection [2], and continuous miniaturization that require an accuracy range in micrometers [3]. Machining tools are also constantly being developed and extended with modern, complex systems, such as compensation of thermal errors of a ball-screw-driven [4] or cutting stability [5,6]. In addition, more advanced solutions have been proposed by Gomez-Acedo et al. [7]. The authors proposed a special method designed for the assessment of repeatability and accuracy of large machine tools, along with results for a large gantry-type milling machine, recorded in a medium-term period. For this purpose, they recorded temperatures, and a metrological frame was used along with inductive sensors in the tool tip, performing repetitive measurements. Laser solutions are also used to compensate for thermal errors in machines. In [8], the authors presented a new methodology to measure thermal distortion in large machine tools based on interferometers. The proposed method used a single tracking interferometer that can be used to measure thermal distortion of machines with large work volumes while maintaining a low enough measurement cadence and uncertainty. Vision systems are also developing very quickly. They are applicable for vision-based 3D scanning system for the positioning of the workpiece [9], and systems based on models for stability prediction [10] or used in advanced applications [11]. Various types of contextual software for CNC machine tools and software used to generate programs based on three-dimensional (3D) models have been introduced, which unfortunately require additional skills in modeling 3D objects and involve the need to purchase additional software. Despite the dynamic development of graphical interface systems [12], overlays for generating G-code and interactive assistants [13,14,15] in CNC systems, the issue of intuitive programming of a CNC machine tool is still not solved. The way CNC machines are controlled has not changed for years and is based on buttons/wheels or writing/generating G-code. Despite all of these systems, errors involved in the assembly machine can still occur. In [16], the authors proposed a methodology for the assessment of the geometrical accuracy of a multiaxis machine.

The inspiration for designing a manual programming system for a CNC machine was developed from programming industrial manipulators [17,18]. The method uses a manual programming tool to move the tip of a manipulator and then gives commands to save individual positions [19]. As a result, it is possible to quickly generate a trajectory between the saved individual points. Based on this outcome, an innovative method of programming a CNC machine tool has been developed and software has been prepared to simulate the programming process in virtual reality [20,21,22,23]. The process of programming manipulators through their manual operation is supported by various types of systems. In such a solution, the operator can intuitively grasp the manipulation system located at the end of the robot arm and move the robot arm by moving the hand [24,25]. This work was further used for manipulator control in milling processes using teach-in methods [26,27] and in the foundry industry to process small- and medium-sized series of castings. Unfortunately, in the case of CNC machine tools there are no such simple and intuitive solutions on the market.

The development of a solution appropriate for machining systems is complicated by the requirement for acquiring and maintaining a desired cutting speed to achieve smooth motion of not only one but several axes of the machine tool at the same time. When operators handle the CNC machine tool they must operate the movement of several axes at the same time. The system for manual handling of the CNC machine tool body parts should also be intuitive, like moving objects in real world—many realistic manual control tasks require human operators to control multiple degrees of freedom simultaneously [28]. As shown in research, humans perform better when controlling multiple axes simultaneously than when they control each axis independently [29,30]. Additionally, in independent axis control (as in buttons, simple joysticks or pads) a focus on one axis or a consistent prioritization has been observed, an effect referred to as axis asymmetry [31]. The sensation of force when moving objects is important for intuitive control [32].

In the literature there are efforts in which a bulk of work is centered mainly upon haptic technology [33,34] or force-feedback [35,36]. In a haptic system with force feedback, only virtual machine tools are controlled, and not physical CNC machines [37]. In [38], results were presented for a programming by demonstration (PbD) interface in augmented reality for motion planning in a three-axis CNC machine. The interface assists a human planner to effectively determine dispenser motion in a planning task. Haptic solutions are also used for the training of machine operators and path planners in virtual polishing and grinding machines. A tool-workpiece contact force model was developed to simulate resultant haptic force feedback [39]. Likewise, there are also several similar solutions in the refurbished conventional machining industry. A simplified version of point saving solutions is used in refurbished conventional machine tools additionally equipped with simple CNC systems. These systems enable manual operation, where the operator controls the machine tool with the aid of handwheels, performing the machining in real time, and in the teach-in mode. While in teach-in mode, the operations performed by the operator during the machining of the first workpiece are recorded. It is possible to repeat the operations and thus perform automatic machining of subsequent parts. The operator-machine interfaces in such solutions are not very intuitive as they are still based on archaic input systems, i.e., handwheels and buttons. Despite all of these achievements in the field, there is no solution for modern CNC machine tools.

In this study we propose an interactive haptic guidance system for the intuitive programming of CNC machine tools. We consider the following to be our key contributions:

- We introduce the intuitive haptic method of controlling CNC machining tools based on the natural moving of objects for simple machining operations. The proposed programming approach allows a machine tool to be programmed without writing program code or knowing specialized software. With this CMGS system, people with no experience, who are not CNC machine operators and who do not know the G-code, will be able to operate the machine.
- We conducted a user study to test proposed solutions. Tests consisted in the comparison of technological operations cloned using the traditional method and the proposed CMGS system. Results show that the proposed system does not increase programming time compared to standard program code development but is easier for the operator.
- We propose an algorithm to calibrate the CMGS system for a specific user, so as to calibrate system parameters to make the machining tool motion most comfortable for each operator.

The outline of the paper is as follows: First, the architecture is presented followed by the concept and implementation of the innovative manual guidance system for the programming of CNC machine tools directly on the machine. Section 2 presents a detailed description of the structure and assumptions of the system. Section 3 contains a description of tensometric control levers and the developed human-machine interface. The experimental validation of the proposed solution is described in Section 4 and conclusions are presented in Section 5.

## 2. CMGS—CNC Manual Guidance System

In this paper, the authors created an approach that relieves the operator from needing fluency in G-code or any other programming language. Therefore, the approach becomes intuitive and allows for faster training of beginner operators. The approach was inspired by the methods of programming industrial manipulators and by using the method of teaching-in. The method of teaching-in stems from the field of robotics and consists in saving all traversed points and settings such that the movement track can be easily reproduced. The application of this method is presented below for the purposes of CNC machine tool operators.

Programming the motion trajectory of a CNC machine tool is a very difficult task as it requires moving two independent kinematic chains. Furthermore, another challenge is to create the process to move the machine in an intuitive way, eliminating mistakes in selecting directions and senses. This requires moving the machine in several axes simultaneously at different speeds and to be able to work closer to the working space of the machine. In order to achieve all these objectives, a new human-machine interface (HMI) had to be created, which makes it possible to enter the necessary data by the CNC operator. The idea was to develop a solution that would allow the machine to be taught by saving individual points in space and related parameters, which could then be used to generate a machining program for the machine tool control system.

The first step to programming the CNC machine tool is to enable the operator to move the body parts of the CNC machine tool to the intended positions. Since CNC machine tools are equipped with electric drives and control systems to maintain a given position, it is not possible to move, e.g., the machining table by pushing it manually. In addition, in the vast majority of cases, CNC machine tools have a lead screw, so switching off the control system and drives also does not allow free movement of the table or headstock. This is because the screw driven from the load side has self-locking properties and the mass of the headstock is in most cases too high to be raised by the average person.

In order to move a machine axis, it is necessary to prepare and deliver the relevant data to the CNC control system and then start the motion procedure with the correct parameters. The control system uses the data to map the position and speed setpoints for the drives. There are different methods of entering motion data (motion commands) to include keyboards, knobs, buttons, touch panels, etc. However, the use of these methods is often inconvenient due to the high probability of making mistakes related to the confusion of directions or senses of movements of individual moving body parts of the machine tool, which can result in dangerous events for the operator and the machine.

**Figure 1:**
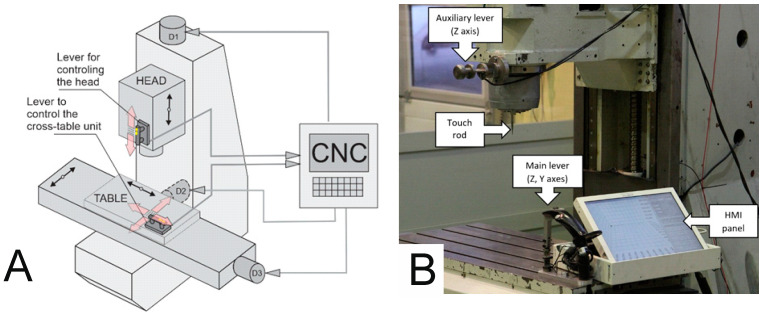
(**A**) Schematic diagram of the CMGS system, (**B**) input interfaces to the CMGS system.

In order to minimize the number of such events and to mitigate their possible consequences, system builders often deliberately restrict the ability of a machine to move, allowing movement on only one axis at a time with some speed constraints. The speed is chosen such that it is limited and safe for the operator to stop the machine and/or force the machine to work at a certain distance from the machine. In order to combat these limitations, a new arrangement of the motion control systems (levers) was adopted, where they are permanently fixed to the end body parts of the CNC machine tool in such a way that their senses and directions coincide with the directions and senses of movement of the machine tool body parts. The ideas of the controllers’ arrangement are presented in Figure 1A.

In the natural human environment, the movement of objects in space is not achieved by holding a button, but by exerting an impact on it with the force of muscles. In this way, a person can move objects at different speeds depending on the force applied. In order to make the operation of the machine as intuitive as possible, the operator can smoothly change the speed when moving the body parts of the machine. This is instead of the conventional on-off “arrow” type buttons. In the natural environment, an operator can move workpieces in different directions and not only along a single axis (and only this possibility is provided by control systems used in CNC machine tools); the approach here is to allow for the developed system to provide the possibility of safe work with a CNC machine tool simultaneously on several axes.

CNC machine tools usually have two kinematic chains, i.e., the object branch and the tool branch. In this work, the AVIA Precision Machine Tool Factory type VC760 (Fabryka Obrabiarek Precyzyjnych AVIA S.A., Warsaw, Poland) was used at the Faculty of Mechanical Engineering and Mechatronics of the West Pomeranian University of Technology in Szczecin. The levers were placed on the end element of the tool branch, which is the head and the end element of the object branch which is the table. The lever on the head is designed to handle one direction (two senses) of the axis from the Cartesian system. The data from this lever are sent to the control system, which develops the drive torque of the electric motors to map the movement of the operator’s hand. The lever mounted on the table works in two directions: *X* and *Y*. When the operator interacts with the lever to control the head, the CNC machine tool control system must be informed in real time so that it has the correct torque for the N1 drive, thus ensuring a certain speed of movement and allowing accurate positioning of the tool. The same is true for the cross-table control lever, which is responsible for the *X* and *Y* axes. The propelling moments are developed on the propelling devices N2 and N3.

### System Architecture

The PXI measurement and control platform developed by National Instruments were chosen as an intermediate platform. This platform has a large selection and configuration of input/output cards and enables processing signals in real time. The PXIe-1082 housing was supplemented with a PXIe-8133 controller module equipped with a CORE i7-820QM 1.73 GHz processor. To measure signals from the strain gauges, we chose a PXIe-4330 card equipped with eight channels, enabling measurements with a resolution of 24 bits. Additionally, in order to support digital inputs and outputs, a PXI-7842R card containing an FPGA (field-programmable gate array) Virtex-5 LX50 was applied. The platform was equipped with the necessary terminals with cables and the PMA-1115 touch screen monitor. Additionally, LabVIEW was used to program the PXI platform. LabVIEW is a graphical language that was used to program the FPGA chip of the PXI-7842R card.

**Figure 2:**
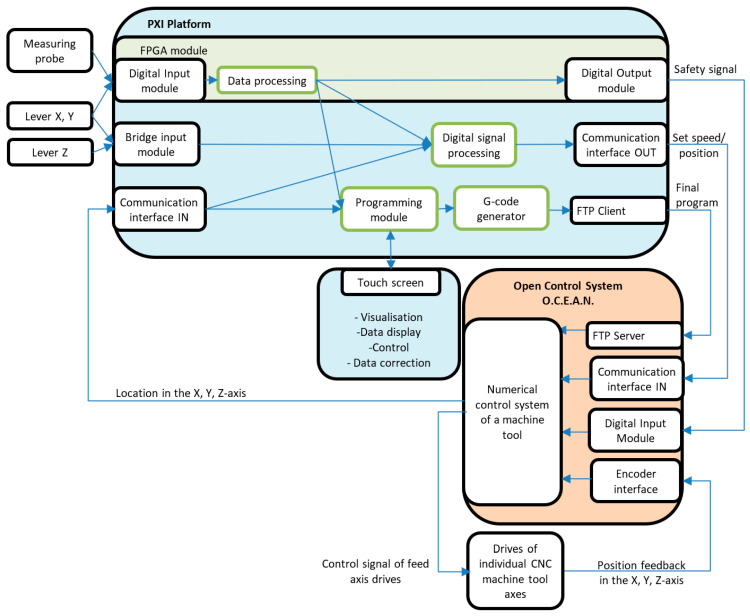
Block diagram of the manual guidance system for CNC machine tool.

A block diagram of the designed system for manual programming of the CMGS is presented in Figure 2. The system consists of input elements (levers and a measurement probe), an input-output element (monitor supporting manual programming), a PXI platform for signal processing and final programming generation, and a CNC machine tool with a control system. The CMGS system is mounted on a three-axis milling machine of medium size of type VC760 produced by Fabryka Obrabiarek Precyzyjnych AVIA S.A. The body system of the machine is equipped with drives with circulating ball feeding screws. The machine has three axes, *X* and *Y* implementing the horizontal movement of the cross-table and *Z* axis implementing the headstock’s vertical movement.

The main control system of the VC760 machine tool drives is the O.C.E.A.N. system. The openness of the system made it possible to implement additional functionalities in order to communicate with the CMGS system. The selected PXI platform is an intermediate element between the levers and the O.C.E.A.N. open control system. The platform communicates with the open control system by exchanging information about the pre-defined position, speed, and acceleration. Based on the measured values and the ones received from the control system, a setpoint speed or position of the axis is generated, which is a setpoint value transmitted to the control system. The setpoints and configuration variables are entered via the touch screen and are used in automatic mode.

To control the cross-table assembly (*X* axis and *Y* axis), the main lever attached to the table is used. To control the position of the head (*Z* axis), an auxiliary lever attached to the headstock is used. The layout of the controls and both levers are shown in Figure 1B.

The main lever has buttons for performing various functions during the programming process. The system is also equipped with a metal rod simulating the simplified form of the measurement probe to perform reference positioning of the workpieces with its action. The application of such a solution was dictated by the fact that the O.C.E.A.N. system did not have standard procedures for handling commercial measurement probes. By means of the probe and data from the measurement system, detection of the probe contact with the measured object is performed. A GUI (graphical user interface) on the touch screen was mounted right next to the main lever so that the operator could directly view data during machine tool control and programming. The touch screen also allows for changing parameters and operating modes. The use of expensive measurement equipment is due to the fact that this is a prototype and the use of specialized equipment and tools allowed for shorter prototyping time. Ultimately, these expensive circuits can be replaced by much cheaper, dedicated electronic circuits integrated in the control lever, which makes economic sense if more prototypes are needed. The prepared dedicated device can be integrated directly with commercial CNC systems.

## 3. Controlling Haptic Devices

During the infant stages in controlling external devices on milling machines, 3Dconnexion used the 3D SpaceNavigator with a Logitech Freedom™ 2.4 Cordless Joystick. 3Dconnexion attached the devices to the CNC machine table for manual control.

**Figure 3:**
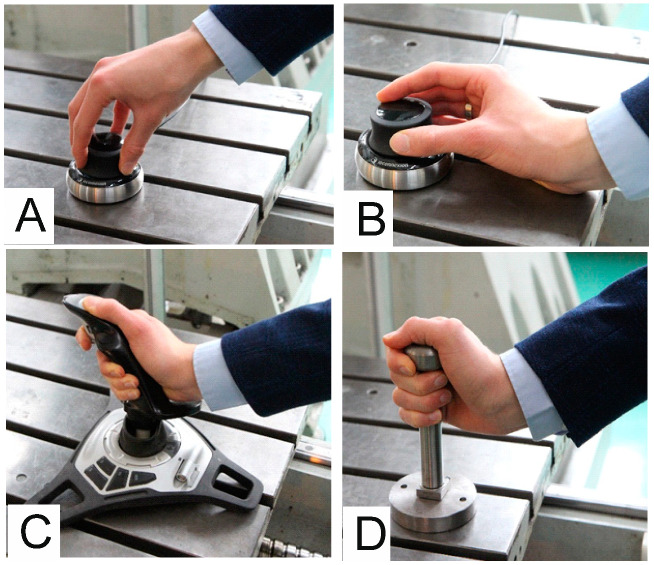
(**A**) 3D SpaceNavigator by 3Dconnexion—Control without wrist rest; (**B**) 3D SpaceNavigator by 3Dconnexion—control with wrist rest; (**C**) Logitech Freedom™ 2.4 Cordless steering through pivoting; (**D**) piezo sensor handle design.

Using the SpaceNavigator as a velocity referencing device to move the machine table presented some challenges due to the high susceptibility of the mouse. It took a small amount of force to swing the handheld mouse knob, which caused large motions for the table. As the table moved, the base of the mouse also moved in the person’s hand. This behavior made it impossible to maintain a constant tilt of the mouse knob relative to its base, and thus a constant table velocity—see Figure 3A.

Improved results were obtained when the person’s wrist was supported on the CNC machine table. This allowed the SpaceNavigator device knob to be tilted with their fingers. Despite allowing for improved control, the control still did not meet the goals of intuitive movement of body parts (Figure 3B). In turn, movement of the machine tool body part was not performed in a way that mimicked moving the part by hand but by moving the fingers relative to the wrist.

With the Logitech Freedom™ 2.4 Cordless joystick, it was possible to change speeds seamlessly. The hand was supported by the joystick handle and the operation of the system depended on the orientation of the clenched hand on the joystick. Changing the orientation changes the angle of the stick with respect to the vertical axis. In this case, the disadvantage of this solution is that the movement of the table and the adjustment of its speed are not dependent on the position but rather on the orientation of the hand (Figure 3C).

During our testing of the levers, we observed that using a manipulator placed directly on the machine eliminates errors associated with moving in the wrong direction and turning away from the intended direction. Tests of commercially available systems have also shown that the manipulator should be stationary, preferably fixed to the controlled element (it must not be able to move freely relative to the measuring system base). Therefore, the design was updated and a third solution was fabricated using a piezo sensor handle (type 9252A) made by Kistler (Figure 3D).

In the case of the designed lever, its handle practically does not deflect with respect to the base, yet the applied force is measured. Initial tests have shown that high stiffness manipulators allow precise control of the movement of CNC machine tool body parts and have clearly indicated the advantage of low susceptibility manipulators with a direct force measurement. In order to reduce the cost of the cuffs, the cuffs were designed with a strain gauge system.

### 3.1. Tensometric Levers

**Figure 4:**
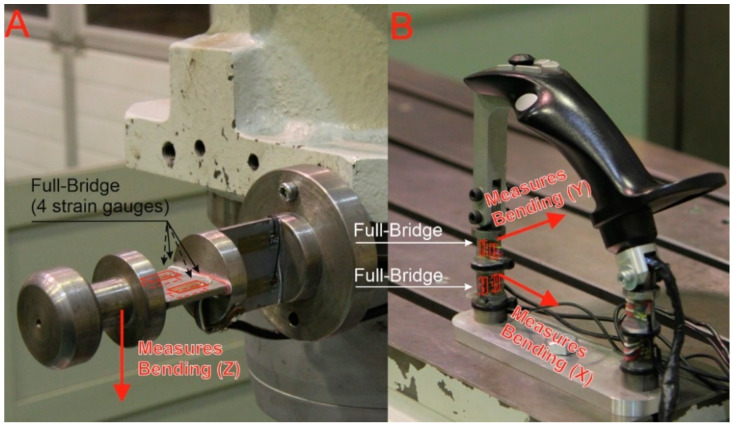
Distribution of strain gauges and neckings: (**A**) Auxiliary lever; (**B**) main lever.

The significant design work centered around the system was to design levers with tensometric force measurement. This system contained two neckings oriented at an angle of 90° to each other, ensuring proper plasticity in both orthogonal directions. The neckings are optimized to provide the required strength while maintaining the specific susceptibility required for correct recording of the strain gauge signals. The location of strain gauges and constrictions is shown in Figure 4.

In the control levers, strain gauges were placed on the lever neckings, as shown in Figure 4. The use of strain gauges in a full bridge system compensates for the influence of external factors, such as temperature.

### 3.2. Signal Processing

A single channel of the PXIe-4330 measuring card is used to measure the bending rate of the string gauge. Data acquisition from the function buttons of the main lever is carried out with the PXI-7842R card. The card is equipped with an FPGA system for data processing and analysis. One of the buttons is the emergency stop signal transmitted directly to the CNC control system. Using a card with an FPGA system allows the user to skip the application layer in the PXI system platform. In this configuration, the safety system is independent of the measuring and programming system, thus shortening the time of communication between the safety system and the control system and therefore increasing the safety of the entire system for manual programming with a CNC machine tool. Additionally, the digital inputs of the card were also used to operate the measuring probe.

**Figure 5:**
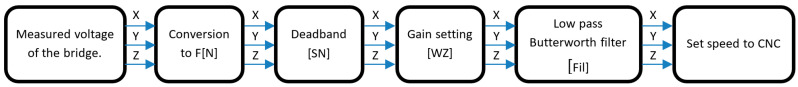
Scheme for converting measuring signals from levers into speed values for the *X*, *Y* and *Z* axes.

**Figure 6:**
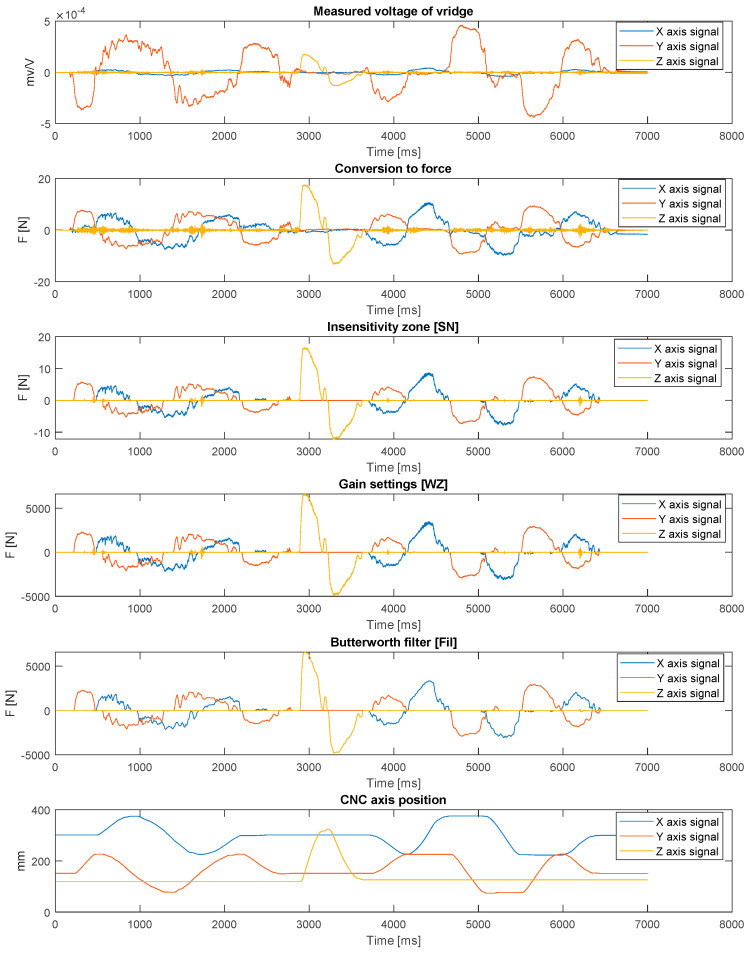
Signals at different stages of the processing algorithm (SN = 2N, WZ = 400, Fil: 4 Hz).

The measurement signal from strain gauge modules is digitally processed to determine the speed at which a given CNC machine tool body element is to move (Figure 5). The final speed depends on the input data to the CMGS from the O.C.E.A.N. system and on the status of the main lever buttons. The signals for the *X*, *Y* and *Z* axes are processed by means of an additional post-processing algorithm whose signals are depicted in Figure 6.

First, the force with which the operator interacts with the lever is calculated as a function of the measured voltage and previous calibration. Another process consists in using a zone of insensitivity to eliminate components of measurement signals with values oscillating around zero, resulting in a continuous pre-setting of movement sense target variables (moments on the motor). The application of this procedure makes it easier to move the table along one axis of the table’s cruciform system, because when moving along one selected axis there are always low signal components from the perpendicular axis, which are cut out by the insensitivity zone. The advantage of this solution is easier operation without the need to switch to single-axis mode. One drawback is the possibility to have a worse representation of circular movements predetermined by the operator. In fact, the operator is not able to perfectly reproduce the circular movement, so one of the basic assumptions of the system is to resign from the possibility of saving the entire track of the movement of the upper limb in favor of saving individual selected and specified points.

At the current stage of system development, the operator can set three basic parameters (SN, WZ and Fil), characterizing the way the machine tool moves. The first one is the insensitivity zone—SN. This parameter allows the operator to set the threshold used to eliminate small values of applied force, which allows for easier guidance on a straight line while making it difficult to guide the tool after curvilinear movement. The WZ parameter is responsible for the signal gain level. This allows the operator to change the level of force that must be applied to the cuff to move the machine tool. The last parameter, Fil, is responsible for setting the low-pass filter. Smaller values allow for a less frequent change of the return while moving the machine tool, which eliminates the system vibrations.

**Figure 7:**
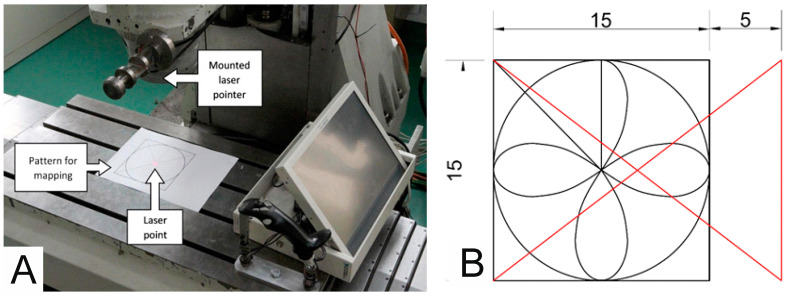
(**A**) Test stand configuration, (**B**) pattern used to calibrate the system.

**Figure 8:**
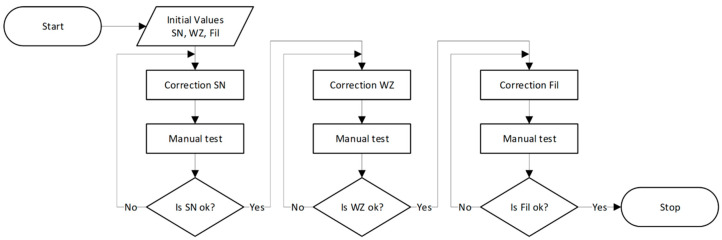
*X*, *Y*, *Z* axes motion path with fourteen different parameter sets T1–T14.

**Figure 9:**
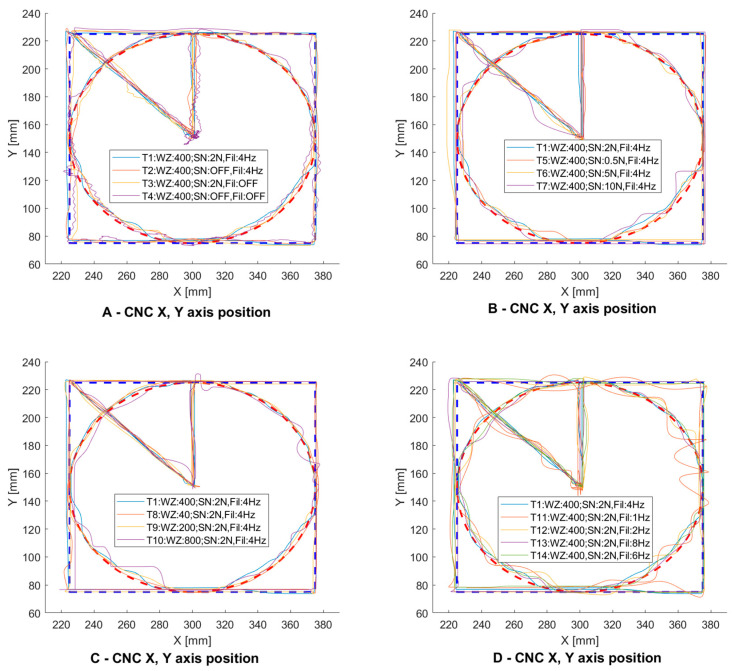
Functional diagram of the parameter (SN, WZ, Fil) selection algorithm for the system operator.

**Figure 10:**
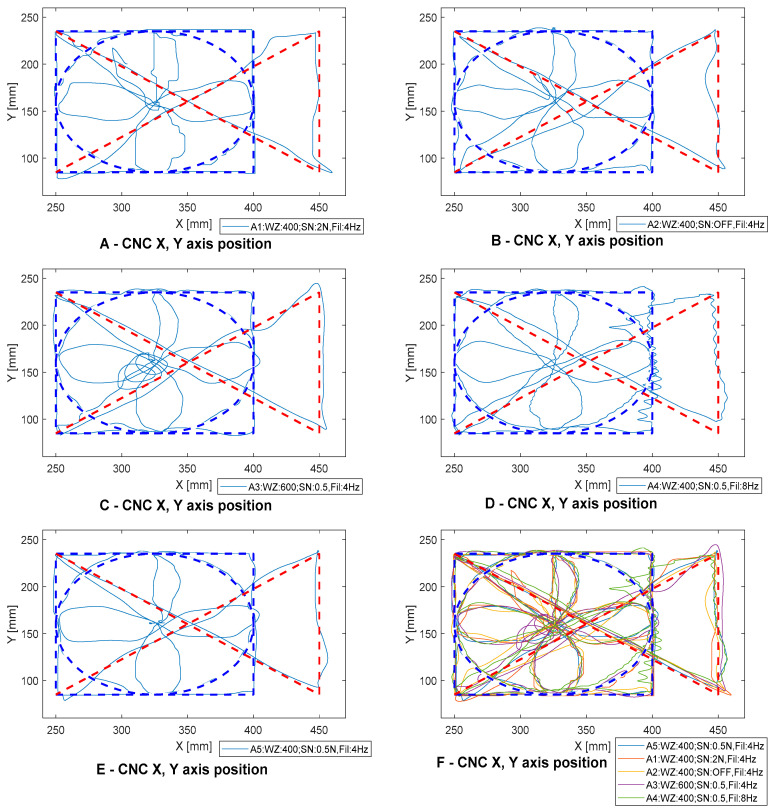
System calibration for first operator.

**Figure 11:**
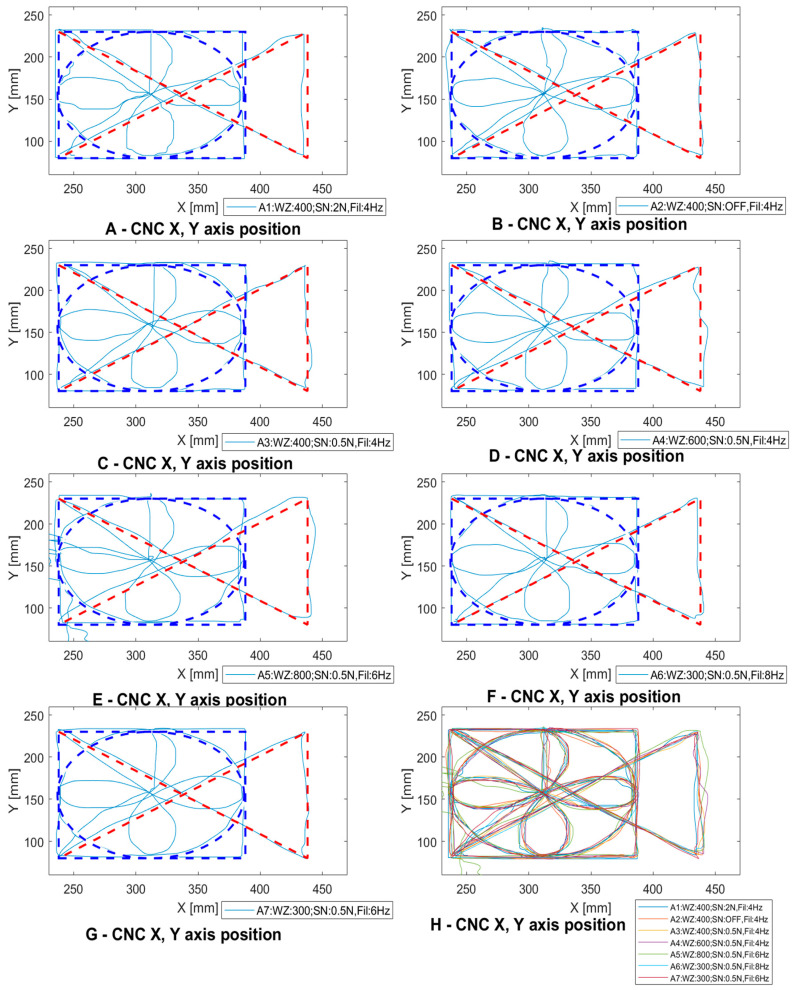
System calibration for second operator.

In order to determine the appropriate values of the parameters (SN, WZ, Fil) of the processing algorithm, 14 tests were carried out. During the tests, the operator had to make moves in accordance with the imposed pattern (Figure 7B; red and blue lines indicate the true path of motion). The current position was indicated by a laser attached to the spindle head. The starting position was in the middle of the circle and movement of the circle was clockwise. The test stand is shown in Figure 7A, the process of setting the WZ, SN, and Fil parameters on Figure 8 and the test results are shown in Figure 9, Figure 10 and Figure 11.

In the first test (Figure 9A, signal T4), a 400 gain was set to ensure that the machine’s body parts could be moved quickly. The signal path did not include a zone of insensitivity. Using these parameters the controlled system oscillated, which made it impossible to precisely position the machine, and the controller was very uncomfortable. Then a low-pass filter was added to remove the oscillations (Figure 9A, signal T2). After adding the filter, a clear improvement in the positioning of the CNC machine tool’s body parts was observed. Since the CNC machine tool control and programming system are used to remember individual points and not the entire mixing route, an insensitivity zone was added. The effect of this change can be observed on the T1 diagram (Figure 9A, signal T1).

In Figure 9B, a change in the parameter of the insensitivity zone can be observed. Increasing the insensitivity zone makes it easier to move the machine only in one axis without unnecessarily inducing motion in the other axes. Setting the insensitivity zone above 5N caused the opposite effect—moving the axis required a high threshold force. Figure 9C depicts a summary of the changes in the amplification parameter. A small amplification allows the machine axis to move at low speed. This allows the operator to reproduce the trajectory more accurately but significantly increases the travel/programming time of the machine tool. On the other hand, too much amplification causes an uncomfortable feeling that the lever is coming out of the operator’s hand. The selection of the low-pass filter parameter is depicted in Figure 8D. Setting the frequency above 4 Hz (6 and 8 Hz) weakened the filter operation. This is due to the frequency with which the operator works. It changes the direction of motion with a frequency of about 4 Hz. Limiting the frequency to 2 and 1 Hz had the opposite effect. The operator had to wrestle with the machine and the response time of the operator was affected, thus causing a significant deviation from the intended trajectory. It should be noted that positioning accuracy was highly dependent on operator concentration and response. Therefore, the parameters in the target system can be changed to match the operator’s preferences.

Each operator has the ability to customize the settings and insensitivity zone reinforcement parameters based on their preferences. The signal characteristics that are presented show how the positioning accuracy of a CNC machine tool changes depending on the settings used. The graphs show the selection of three parameters: low-pass filter (Fil), insensitivity zone (SN) and gain (WZ). The algorithm shown in Figure 9 has been developed to adjust the parameters.

When the system is started, the parameters are set to default initial values. The operator first changes the parameters of the insensitivity zone and then performs the manual guide operation test. This test is repeatable in the case of improper operation. After accepting the settings, the operator goes to the WZ and Fil parameter settings and performs the test. In the future, this process can be attempted to be automated by developing a system that learns how to adjust the parameters to the behavior of the operator recorded during several characteristic movements. The operators have carried out the process of setting the WZ, SN, and Fil parameters in accordance with the procedure presented in the Figure 8.

The process of selection of settings for the first operator is shown in Figure 10. First, tests were carried out on the initial settings WZ400, SN2.0 and Fil 4 Hz (Figure 10A). Then the insensitivity zone parameters were selected. Figure 10B shows the sequence with the insensitivity zone switched off. The operator selected a value of 0.5 for SN and 400 for WZ. Next, the Fil parameter was selected—Figure 10D shows its value of 8 Hz—a clear deterioration of the course is visible, which suggests that using a low-pass filter is necessary in a developed machine tool control system. Final settings preferences for the operator were WZ = 400, SN = 0.5 and Fil = 4 Hz (Figure 10E). It can be seen that the best pattern was obtained for these settings.

The second operator selected and used the following initial settings: WZ = 400, SN = 2.0 and Fil = 4 Hz (Figure 11A). Next, the SN parameter was selected, which is depicted in Figure 11B for the course for the switched off insensitivity zone. It can be seen that it caused a noticeable deterioration of shape reproduction on straight lines. The operator finally set the SN parameter to 0.5 (Figure 11C). In the following approaches whose results are depicted in Figure 11D,E, the gains were changed by 600 and 800, respectively. This caused a deterioration in the pattern mapping. Ultimately, the operator selected the WZ parameter to be 300, which eliminated the deterioration. Next, the different frequencies for the Fil parameter settings were examined. In the case of the second operator, the changes did not cause any significant deterioration in the mapped trajectory (Figure 11F). The preferred settings for the operator are depicted in Figure 11G and were 300 for WZ, 0.5 for SN and 6 Hz for Fil.

The calibration procedure and the mapped shape depend to a large extent on operator preference, speed of movement, and level of concentration. The above study was carried out to illustrate these relationships. The parameter setting process was designed to allow the operator to move the machine’s axes efficiently. To program the points, the operator uses an additional button located on the main joystick. It allows the machine to move precisely with an accuracy of up to 1 µm.

### 3.3. Developed Human-Machine Interface

**Figure 12:**
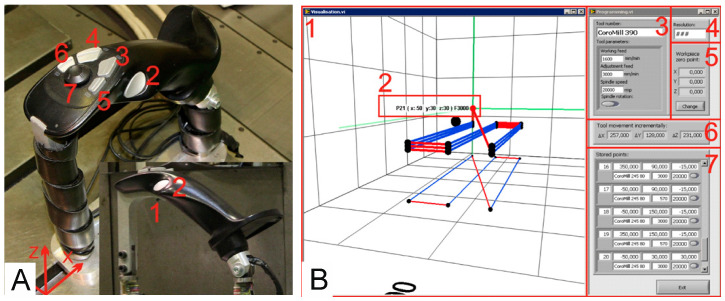
(**A**) Description of the function buttons of the main lever: 1. Safety button (when pressed, it enables the machine to move); 2. slowing down the motion of the machine; 3. saving positions; 4. deleting positions; 5. changing the motion axis; 6. changing the feed rate of the programmed track (working feed/changeover feed); 7. four-component controller for discrete movement on the *X* and *Y* axes. (**B**) Subprogram window for manual programming of a CNC machine tool: 1. Visualization window for displaying the saved track; 2. tool position in the object system along with speed; 3. tools and their parameters; 4. change of accuracy; 5. start of the object system; 6. additional display of incremental tool displacement; 7. saved points with parameters.

The designed operator-CNC machine interface of the CMGS system consists of a main and auxiliary lever and a touch screen monitor (Figure 12B). The main lever is equipped with function buttons that are marked in Figure 12A.

Buttons (2,5,7) are buttons for easy machine control, buttons (3,4,6) take part in the programming process, and button (1) is a safety button. The function of the safety button is as follows: as long as the button is pressed, the machine can be operated. Releasing the safety button immediately stops all machine drives. Using the CMGS system, the body parts of the machine tool can be moved very precisely, especially after pressing button (2), which reduces the speed of the table movement with the same force impacting the lever. The table can also be moved discretely in four directions using the mini joystick—a four-component discrete controller located at the top of the lever (7). The mini joystick can trigger the function for sending information to the data frame control system No. 1. The function has been configured so that the operator can move the table by 0.001, 0.01 or 1 mm depending on the mode selected on the touch screen. Owing to proper processing of measured signals and correct design assumptions of the CMGS system, it is possible to obtain accuracy expressed in single micrometers. Button (3) is used to save the current position of the CNC machine tool body parts with the pre-set feed rate and button (4) is used to delete the last saved position. The current feed rate to be assigned to a given position of the machine tool (working or adjustment feed) can be changed with button (6) on the lever. Button (5) can switch the mode of controlling all axes to the mode of controlling only one axis. The graphical user interface (Figure 12B) is implemented on a touch screen monitor located directly at the main lever, thus enabling comfortable operation.

## 4. Experimental Validation

In order to verify and test the developed system, tests were carried out on a laboratory station. First, the operations of the fully assembled system were checked. Next, tests were carried out to verify its functionality. These tests consisted in the comparison of technological operations cloned using the traditional method and the CMGS system. In the course of the study, the timings of performing individual operations were recorded.

The experimental research program of the CMGS system for manual programming of a CNC machine tool was designed in such a way as to check its functionality on the basis of several representative technological operations, which are widely used in industrial practice. The planned experimental research program consists of six variants of technological operations, three of which are related to the machining of the workpiece and three to its measurement:

1. Technological operations of workpiece machining:
  - Plane face milling—variant I;
  - Milling of pocket cavity—variant II;
  - Through groove milling with drilling of holes—variant III.
2. Technological operations of measuring the workpiece:
  - Measurement of the position of the reference positioning system of the object by measuring three points on mutually orthogonal surfaces in the 1/1/1 system—variant IV;
  - Measurement of the position of the reference positioning system of the object by measuring six points on mutually orthogonal surfaces in the 3/2/1 system—variant V;
  - Measurement of the element diameter by measuring four points—variant VI.

**Figure 13:**
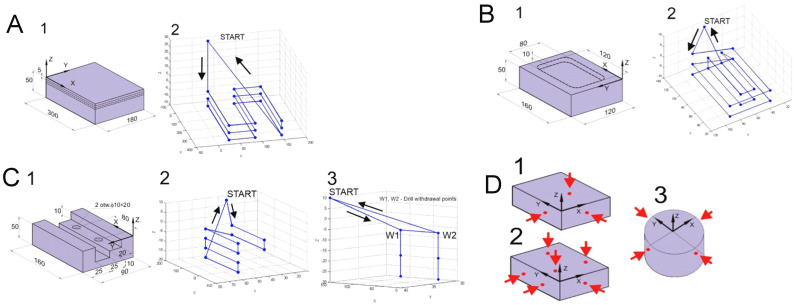
(**A**) Variant I of the study: 1—workpiece to be machined; 2—drawing of the designed tool path milling. (**B**) Variant II of the study: 1—workpiece to be machined; 2—drawing of the designed tool path milling. (**C**) Variant III of the study: 1—workpiece to be machined; 2—drawing of the designed tool path milling; 3—drawing of the designed tool path drilling. (**D**) Diagram of workpiece measurement operation: 1—measurement of workpiece position by 3 points 1/1/1 diagram—variant IV; 2—measurement of workpiece position by 6 points 3/2/1 diagram—variant V; 3—measurement of cylinder diameter by 4 points—variant VI.

The first variant of the test was designed in such a way that the operator had to perform a series of actions, which would result in a programmed rectangular plate machining path. Machining consists of face milling a plate in three passages with a cutting depth of 5 mm. The dimensions of the workpiece, the adopted reference system, and the material to be machined are shown in Figure 13A. CoroMill 245 with a diameter of 80 mm and catalogue number R245-080Q27-12M was chosen as the machining tool. Before the tests, the tool’s track was designed, and it is presented in Figure 13A.

The second variant of the study included milling of pockets in a rectangular plate. First, the external shape of the pocket should be selected (in two passages, each 5 mm deep) and then the material should be removed in the middle of the cavity (also in two passages, 5 mm deep). The dimensions of the workpiece, the adopted reference system, and the shape of the target workpiece after the selection of the material are shown in Figure 13B. The planned track of the tool with characteristic points is presented in Figure 13B. In this case, the cutting tool was the CoroMill 390 with a diameter of 25 mm and catalog number R390-025B25-11M. It allows for a smooth penetration into the workpiece during milling, which is why the technological operation was used based on the tool entering along one of the sides of the edge of the produced pocket. The radius of the pocket curve is determined by the diameter of the cutter.

The third variant of the study included programming the tool and drill track in order to obtain an object with dimensions of 160 mm × 90 mm × 50 mm, as presented in Figure 13C. The figure, apart from the dimensions of the workpiece, as well as the shape and the holes to be made, has the adopted reference system. The task must be performed by means of two technological operations, namely milling and drilling. The first step is to program a track for the through groove and the second step is to program a track for drilling blind holes. The planned path of the cutting tool for the machining of the through groove is presented in Figure 13C. A CoroMill 390 cutter with a diameter of 25 mm and catalog symbol R390-025B25-11M was selected for the machining process. Figure 13C represents the designed tool path for blind hole drilling. A CoroDrill R840 bit with a diameter of 10 mm and catalog number R840 1000 30-A0A 1220, which is a solid carbide drill, was chosen for the drilling operation.

Variants no. IV–VI of the study included the performance of technological operations related to the measurement of the workpiece. This was done by measuring the points that can be used to determine the base coordinate system of the workpiece. A schematic summary of the individual variants is presented in Figure 13D.

The timings of performing operations recorded during the study were compared with those for the same variants of CNC machine tools available for commercial use and operated by an experienced operator using the interfaces provided by the manufacturer.

These operations were performed on the DMU60 monoBlock milling machine with Heidenhain iTNC530 control. In addition to manual input of track points in the programming process, machining cycles were also used. These were the 230 cycles for multifaceted milling and the 251 cycles for rectangular pocket execution. Study variants IV, V and VI were carried out using a commercially available CNC control on a DNU60 monoBlock milling machine using this Heidenhain measuring probe. A wired remote control was used to control the movements of the machine tool. Table 1 presents a summary of the timings of performing individual operations for both compared systems. Before programming begins, it is necessary to define the initial parameters, such as the geometry of the blank, file names, etc. On the DMU60 it took about 3 min 15 s. In the case of a machine tool with the CMGS system for variants I, II, III it took 27, 24, and 55 s, respectively. Shorter time of preparing the initial data in the CMGS system resulted from the lack of necessity to define the parameters of the blank. The number of operations necessary to perform in the DMU machine system is greater and requires proficiency in operating the machine. The CMGS system has greatly simplified the programming procedure.

## 5. Conclusions

Based on the results of this effort that are summarized in Table 1, it can be concluded that manual programming using the CMGS system and programming using commercially available control systems are similar if the operator does not use machining cycles. It is worth noting that manual programming can be achieved much faster if the operator/programmer does not have to precisely reach the points specified in tests in real-life conditions, where the tolerance range of some characteristic points is quite large, e.g., the point where the tool goes beyond the contour of the workpiece. From the machining point of view, it does not matter whether the point is 30 or 30.45 mm away from the workpiece, but the need to set the exact value significantly slowed down the manual programming process, which required tracking all the coordinates displayed on the monitor. In the next stage of system development, programming time can be shortened by changing the form of information presentation and by using wizards appearing on the screen while making moves, suggesting subsequent program points based on previous ones, which would require validation only by pressing a button on the lever.

The results obtained during the measurements of the workpiece confirm the earlier thesis that the measurements carried out with the use of manual control using the CMGS system had a much shorter duration than the measurements carried out on a commercially available CNC machine tool, DMU60 monoBlock.

The results suggest that the use of machining cycles accelerates the commercial machine programming process while extending the time needed to prepare the initial data due to the need to define the geometry of the blank. In the future, the functionality of the CMGS system may also be enriched with the support of machining cycles. The conducted research proved that manual programming simplifies the programming procedures of a CNC machine tool. Manual programming is simple and intuitive because moving the machine’s body parts is similar to moving objects in the world around us.

As a result, the methodology depicted here was able to accommodate confusing directions and senses during testing, even when moving three axes at once. This feature of the system allows for a significant acceleration of the workpiece measurement process. Determining a workpiece’s coordinate system is simple and quick to master. For the programming process itself, in most cases the functionality of saving the position and changing the feed rate was sufficient.

Other functions and the required pressing of buttons to activate them, as well as the options available on the display, provide additional support. The machine tool can be moved reliably and error-free with variable speed depending on the applied force in accurate mode with high resolution. As a result, the CMGS system allows to generate machining programs with the desired accuracy, without significantly increasing the programming time.

The use of the prepared solution relieves the operator of the need to manually write programs and know the programming languages of the machine. The designed, built and programmed CMGS system has a significant potential to expand and implement many programming procedures and even design spatial objects on the machine tool.

The CMGS system prototype presented in the paper can be effectively used for programming selected technological operations. In the case of repair and prototype workshops and companies that produce simple parts in small batches, there is a need to quickly perform simple technological operations on the machine tool, such as face milling, groove milling, choosing a simple pocket or drilling holes. Creating a CAD model for a simple object can take more time than machining, so using CAD/CAM software can be unreasonable and inefficient. Often, conventional machine tools without CNC control are used because they do not require writing programs, let alone CAD/CAM software application. Their capabilities are often sufficient for the current production.

Equipping a CNC machine tool with a manual programming system can increase its functionality and usefulness by allowing easy manual control and programming by a less experienced operator, thus adding to an advanced CNC machine tool the functionality and simplicity of a conventional machine tool.

Our key contributions are:

- We introduced the intuitive haptic method of controlling CNC;
- We conducted a user study to test, which confirmed the effectiveness and ergonomics of the proposed solution;
- We proposed an algorithm to calibrate the CMGS system for a specific user in the prototype phase, which showed encouraging results.

## Figures and Tables

**Table 1:** Results of tests of functionality of the manual programming system using the CMGS system and commercially available Heidenhain iTNC530 control system.

Study Variant No.	Implementation Time		
	Heidenhain iTNC530		Manual with CMGS Levers
	Without Cycle	With Cycle	
Machine tool programming			
Variant I—surface milling	4 min 55 s	1 min 10 s	4 min 8 s
Variant II—cavity milling	6 min 25 s	3 min 12 s	7 min 40 s
Variant III—groove milling	6 min 35 s	-	8 min 14 s
Measurement of a workpiece			
Variant IV—1/1/1 measurement	1 min 2 s	-	12 s
Variant V—3/2/1 measurement	1 min 10 s	-	13 s
Variant VI—cylinder measurement	45 s	-	10 s

## Data Availability

Contact with correspondence author.
